# Endoscopic ultrasound-guided duodenojejunostomy for recurrent malignant duodenal obstruction after surgical gastrojejunostomy

**DOI:** 10.1055/a-2928-4382

**Published:** 2026-09-23

**Authors:** Sung Woo Ko, Seung Bae Yoon, Inho Lee, Sung Woo Hyung, Hyehong Min, Ju Young Park

**Affiliations:** 1 Department of Internal MedicineEunpyeong St. Mary’s Hospital, College of Medicine, The Catholic University of KoreaSeoulKorea; 2 Department of Anesthesiology and Pain MedicineEunpyeong St. Mary’s Hospital, College of Medicine, The Catholic University of KoreaSeoulKorea; 3 Outpatient Nursing TeamEunpyeong St. Mary’s HospitalSeoulKorea

Endoscopic ultrasound-guided gastroenterostomy (EUS-GE) is an established minimally invasive treatment for malignant gastric outlet obstruction 1 . Endoscopic ultrasound-guided duodenojejunostomy (EUS-DJ) has been reported as an alternative option when EUS-GE or enteral stenting is not feasible 2 3 4 . Here, we present a case of recurrent malignant duodenal obstruction after prior surgical gastrojejunostomy successfully managed with EUS-DJ.

A 47-year-old man with metastatic pancreatic tail cancer presented with persistent vomiting. He had previously undergone laparoscopic gastrojejunostomy for malignant duodenal obstruction. Despite surgical bypass, progressive tumor invasion of the distal duodenum and narrowing of the anastomosis resulted in recurrent obstructive symptoms. Duodenal stenting was attempted but failed due to a tight stricture ( Fig. 1 ).

**Fig. 1:**
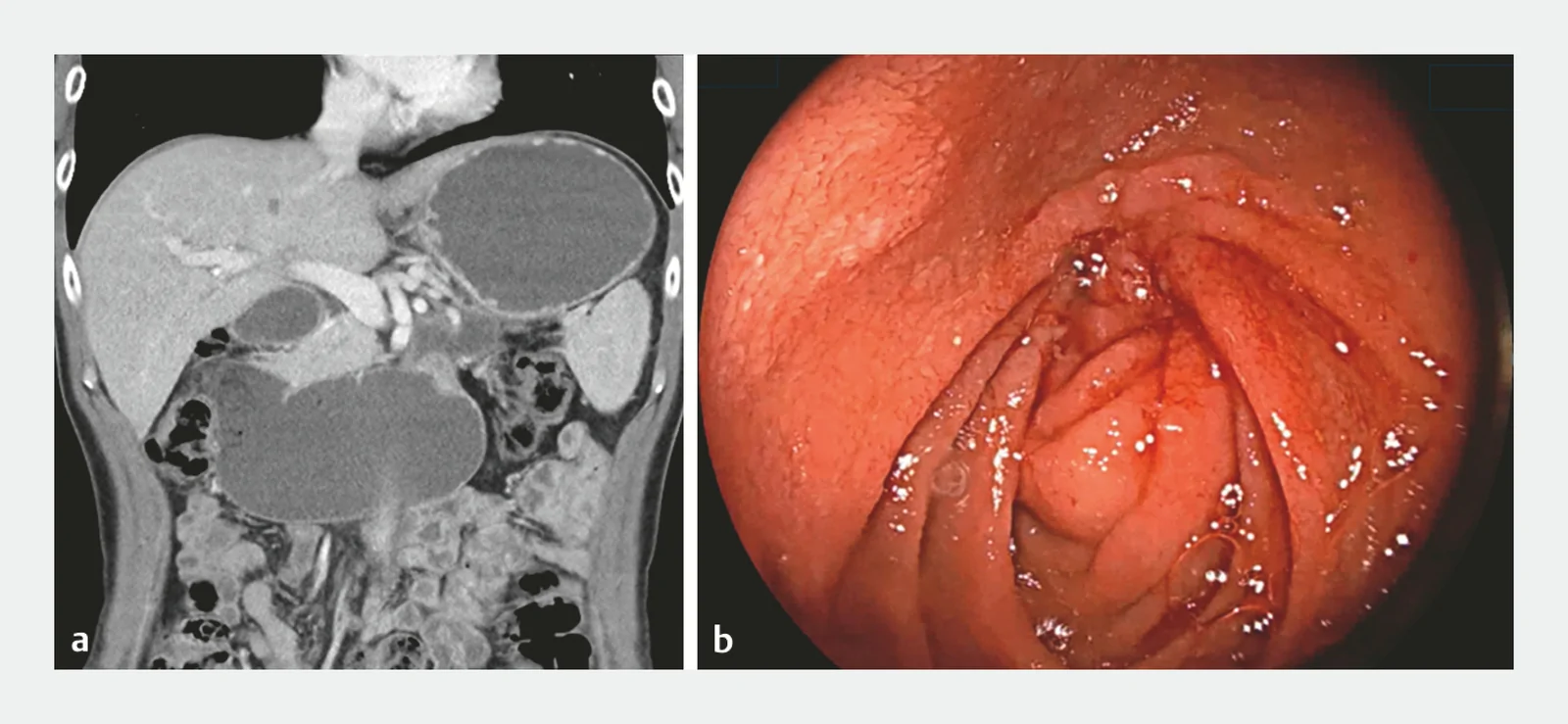
**a** Contrast-enhanced computed tomography showing marked gastric dilatation with obstruction of the distal duodenum. **b** An endoscopic view demonstrating a tight malignant duodenal stricture with near-complete luminal obstruction.

Fig. 2 illustrates the schematic overview of the procedure. Under general anesthesia with nasotracheal intubation, a pediatric colonoscope (PCF-H290; Olympus, Tokyo, Japan) was advanced through the surgical gastrojejunostomy into the efferent jejunal limb. A 0.025-inch guidewire was inserted deeply into the jejunum, and a 7-Fr nasobiliary catheter (Cook Medical, Bloomington, IN, USA) was advanced over the wire. After the withdrawal of the colonoscope, a mixture of saline, indigo carmine, and contrast was injected through the nasobiliary catheter to distend the jejunal loop.

**Fig. 2:**
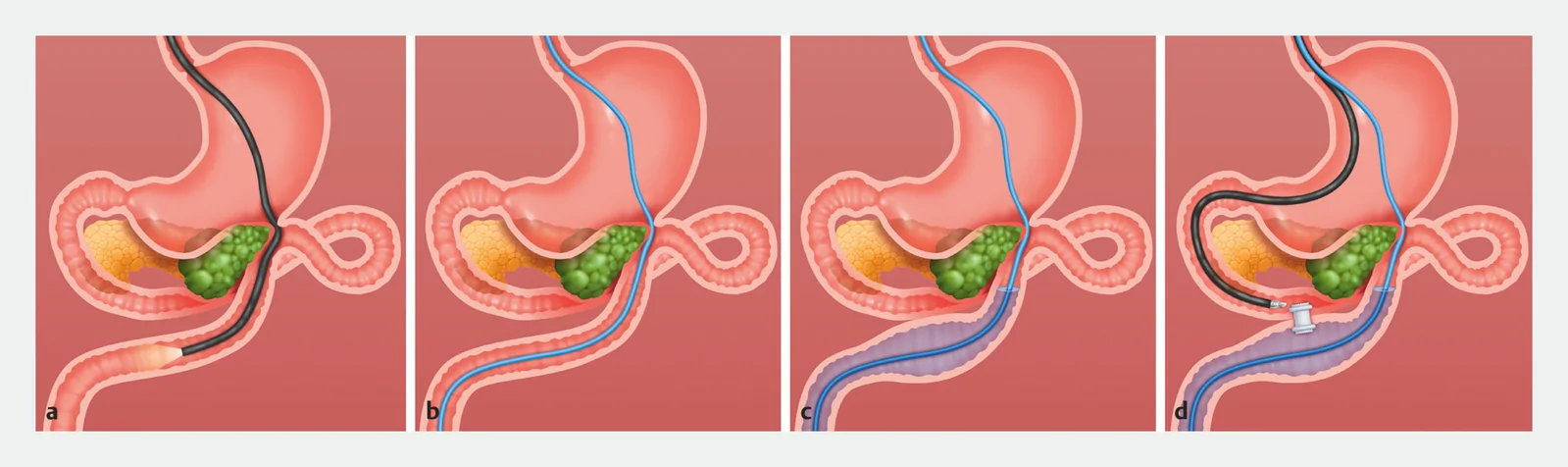
Schematic illustration of endoscopic ultrasound-guided duodenojejunostomy (EUS-DJ). **a** Tumor invasion causes narrowing of the surgical gastrojejunostomy, resulting in impaired luminal passage. A pediatric colonoscope is advanced through the surgical gastrojejunostomy into the efferent jejunal limb. **b** A guidewire is inserted into the jejunum and a 7-Fr nasobiliary catheter is advanced over the guidewire. **c** A mixture of saline, contrast, and indigo carmine is injected to distend the jejunal loop. **d** A linear echoendoscope is advanced into the duodenum and EUS-DJ is performed using a lumen-apposing metal stent [rerif].

A linear echoendoscope (GF-UCT260; Olympus, Tokyo, Japan) was then advanced into the duodenum. On endoscopic ultrasound (EUS), the distended jejunal loop was clearly visualized. A cautery-enhanced lumen-apposing metal stent (HOT AXIOS, 15 × 10 mm; Boston Scientific, Marlborough, MA, USA) was deployed under EUS and fluoroscopic guidance to create a duodenojejunostomy ( Video 1 ). Immediate efflux of blue-stained fluid confirmed successful anastomosis. Two days after EUS-DJ, an upper gastrointestinal series confirmed contrast passage through the duodenojejunostomy ( Fig. 3 ). The patient resumed oral intake and was discharged without recurrent symptoms.

EUS-guided duodenojejunostomy using a cautery-enhanced lumen-apposing metal stent for recurrent malignant duodenal obstruction after surgical gastrojejunostomy. EUS, endoscopic ultrasound.Video 1

**Fig. 3:**
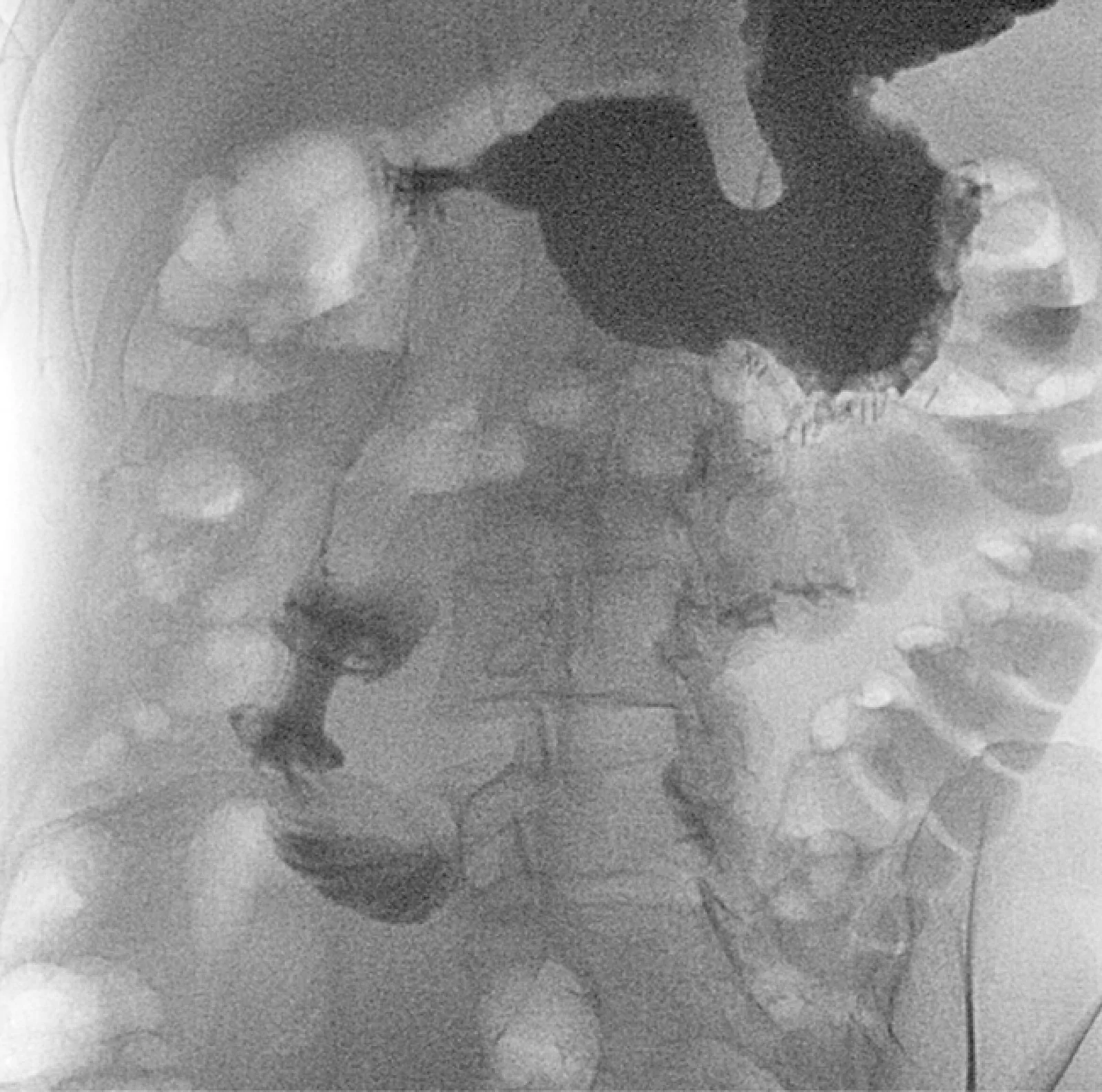
Upper gastrointestinal series performed 2 days after EUS-DJ demonstrating smooth contrast passage through the duodenojejunostomy. EUS-DJ, endoscopic ultrasound-guided duodenojejunostomy (EUS-DJ).

This case highlights that EUS-DJ may serve as a feasible rescue option for recurrent malignant duodenal obstruction after prior surgical gastrojejunostomy when enteral stenting is not feasible.

Endoscopy_UCTN_Code_TTT_1AS_2AK

Citation Format

Endoscopy 2026; 58: E633–E634. DOI: 10.1055/a-2868-7927

## References

[1] Bang JY Puri R Lakhtakia S Endoscopic or surgical gastroenterostomy for malignant gastric outlet obstruction: a randomised trial Gut 2025 75 24 32 10.1136/gutjnl-2025-336339 40998416 PMC12703243

[2] Donatelli G Cereatti F Spota A Long-term placement of lumen-apposing metal stent after endoscopic ultrasound-guided duodeno- and jejunojejunal anastomosis for direct access to excluded jejunal limb Endoscopy 2021 53 293 297 10.1055/a-1223-2302 32767287

[3] Lajin M Catalano MF Orr CE EUS-guided duodenojejunostomy by use of a 2-cm lumen-apposing metal stent to treat proximal jejunal stricture in a patient with chronic pancreatitis VideoGIE 2020 5 75 76 10.1016/j.vgie.2019.11.004 32051915 PMC7004921

[4] Nie S Wang L Shen S Endoscopic ultrasound-guided duodenojejunostomy for a duodenal duplication cyst presenting with duodenal obstruction VideoGIE 2026 11 16 20 10.1016/j.vgie.2025.08.008 41584624 PMC12826998

